# A scoping review of core outcome sets and their ‘mapping’ onto real-world data using prostate cancer as a case study

**DOI:** 10.1186/s12874-020-00928-w

**Published:** 2020-02-27

**Authors:** Michela Meregaglia, Oriana Ciani, Helen Banks, Maximilian Salcher-Konrad, Caroline Carney, Sahan Jayawardana, Paula Williamson, Giovanni Fattore

**Affiliations:** 1 CERGAS, SDA Bocconi, Milan, Italy; 2 Institute of Health Research, University of Exeter Medical School, Exeter, UK; 3 LSE Health, London School of Economics, London, UK; 4 MRC North West Hub for Trials Methodology Research, Department of Biostatistics, University of Liverpool, Liverpool, UK; 5 Department of Social and Political Sciences, Bocconi University, Milan, Italy

**Keywords:** Core outcomes set (COS), Core outcome measures in effectiveness trials (COMET), Prostate cancer, Real-world data (RWD), Mapping

## Abstract

**Background:**

A Core Outcomes Set (COS) is an agreed minimum set of outcomes that should be reported in all clinical studies related to a specific condition. Using prostate cancer as a case study, we identified, summarized, and critically appraised published COS development studies and assessed the degree of overlap between them and selected real-world data (RWD) sources.

**Methods:**

We conducted a scoping review of the Core Outcome Measures in Effectiveness Trials (COMET) Initiative database to identify all COS studies developed for prostate cancer. Several characteristics (i.e., study type, methods for consensus, type of participants, outcomes included in COS and corresponding measurement instruments, timing, and sources) were extracted from the studies; outcomes were classified according to a predefined 38-item taxonomy. The study methodology was assessed based on the recent COS-STAndards for Development (COS-STAD) recommendations. A ‘mapping’ exercise was conducted between the COS identified and RWD routinely collected in selected European countries.

**Results:**

Eleven COS development studies published between 1995 and 2017 were retrieved, of which 8 were classified as ‘COS for clinical trials and clinical research’, 2 as ‘COS for practice’ and 1 as ‘COS patient reported outcomes’. Recommended outcomes were mainly categorized into ‘mortality and survival’ (17%), ‘outcomes related to neoplasm’ (18%), and ‘renal and urinary outcomes’ (13%) with no relevant differences among COS study types. The studies generally fulfilled the criteria for the COS-STAD ‘scope specification’ domain but not the ‘stakeholders involved’ and ‘consensus process’ domains. About 72% overlap existed between COS and linked administrative data sources, with important gaps. Linking with patient registries improved coverage (85%), but was sometimes limited to smaller follow-up patient groups.

**Conclusions:**

This scoping review identified few COS development studies in prostate cancer, some quite dated and with a growing level of methodological quality over time. This study revealed promising overlap between COS and RWD sources, though with important limitations; linking established, national patient registries to administrative data provide the best means to additionally capture patient-reported and some clinical outcomes over time. Thus, increasing the combination of different data sources and the interoperability of systems to follow larger patient groups in RWD is required.

## Background

In recent years, there has been a rapid acceleration in the use of real-world data (RWD) in clinical research and practice. From the perspective of the European Medicines Agency (EMA), RWD are defined as “routinely collected data relating to a patient’s health status or the delivery of health care from a variety of sources other than traditional clinical trials” such as electronic medical/health records (EMRs/EHRs), claims data, prescription data, and patient registries [1]. The United States (US) Food and Drug Administration (FDA) reports a similar definition [2]. Among these sources, longitudinal databases, and especially EHRs, provide detailed records for high numbers of patients, and they continue to grow in size, clinical detail, and accessibility through data linkage, standardization, and sharing. However, several limitations arise when using these sources for the evaluation of effectiveness and safety of health interventions, including heterogeneity of reported outcomes, non-standardized measurements, and inconsistencies across different databases [3, 4]. Despite the growing use of real-world evidence to support broader use of effective therapies and to contribute useful information about treatment effectiveness, just because RWD exist does not mean that those data will be useful for every research question. The utility of RWD data can generally be improved by understanding how well available data characterizes the outcomes of interest, recognizing that information recorded in structured fields are easier to find and analyse than unstructured notes, which may not even be accessible to researchers [5].

In recent times, various groups of trialists around the world and in different disease areas have made efforts to agree on standardised outcomes and their measurement across studies. As a result of their research effort, core outcome sets (COS) have been defined as minimum sets of outcomes which should be measured and reported in all clinical trials of a specific disease condition or for application in other contexts (e.g., disease registries or clinical practice) [6]. Particularly with the rising use of RWD for research purposes, the importance of COS extends now beyond the realm of clinical trials. The selection of a ‘good’ COS is not straightforward, and a quality evaluation process has become essential to discriminate among the growing number of COS development studies. The Core Outcome Set-STAndards for Development (COS-STAD) encourage researchers to comply with minimum standards for COS development and to help users assess whether a COS should be adopted in practice [7].

This study relied on the publicly available and routinely updated electronic database maintained by the Core Outcome Measures in Effectiveness Trials (COMET) Initiative, which promotes the development and application of COSs on a wide range of disease areas [6]. Based on the COMET database, we aimed to identify, summarize, and critically appraise a group of published COS development studies, and to assess the degree of overlap between the identified COS and existing RWD sources through a ‘mapping’ exercise.

The current study was conducted as part of the coordination and support project, DO-IT (http://bd4bo.eu/index.php/portfolio/do-it/), for disease-specific Big Data for Better Outcomes (BD4BO) projects, part of the Innovative Medicines Initiative 2. Among the disease areas covered by these projects, we identified prostate cancer as a relevant condition to address the aim of this study. Prostate cancer is the most common malignancy among males worldwide; more than 1 million cases are diagnosed annually, and the number of deaths has risen to over 300,000 per year [8, 9]. Although survival remains a key outcome in studies evaluating novel therapies, patient-reported outcomes (PROs) are increasingly used in prostate cancer trials and to monitor real-life consequences of a treatment and effectiveness in everyday clinical practice. A PRO is any report of the status of a patient’s health condition that comes directly from the patient or in some cases from a caregiver or surrogate responder, without interpretation by a practitioner or anyone else [10]. The most common patient-reported outcome measures (PROMs) in prostate cancer are the Expanded Prostate Cancer Index Composite (EPIC) and the Functional Assessment of Cancer Therapy – Prostate (FACT-P) questionnaires [11].

## Methods

In order to address the aims above, we conducted a scoping review of COS development studies in prostate cancer, including a quality assessment and mapping of recommended outcomes onto RWD sources. In detail, we first identified which COS studies were available in prostate cancer and which outcomes and outcome measurement instruments (OMIs) they recommended. Second, we verified whether the existing COS studies were developed according to the minimum methodological COS-STAD standards. Lastly, we empirically tested to what extent the measurement of COS in RWD sources is possible and provided insight on how to improve real-world collection of standard outcomes and measures useful for the assessment of healthcare interventions.

This study followed the PRISMA Extension for Scoping Reviews (PRISMA-ScR) [12]. No published protocol is available for this scoping review.

### Studies identification and data extraction

COS development studies designed for different purposes (i.e., clinical trials and clinical research, clinical practice, patient reported outcomes), as defined by the COMET Initiative, were identified by searching the COMET database using “prostate cancer” as the disease name (last accessed: June 2018) [6]. An update of the database is conducted yearly using a systematic approach, originally described in Gargon et al. [13], to maintain the currency of database content [14]; therefore, no additional literature searches were conducted.

We excluded unpublished studies (or studies not published in peer-reviewed journals), studies classified differently from ‘COS studies’ (e.g., ‘recommendations’, ‘definitions’ or ‘literature reviews’) and duplicate studies. A pilot-tested extraction was performed using a few studies, and thereafter a final template was generated in Excel® to collect detailed information in a standardized manner from the studies, identify the methods for consensus and gain specific knowledge of the structure and content of the COS proposed. The template was organized around four broader themes (i.e., study information, study type, methodology for COS development, COS description). A newly developed 38-item scale for outcome classification [15] was used to categorize the outcomes forming the COS presented by the included studies. We used cross-tabulation methods to synthesise the rich information gained from the studies.

### Assessing the quality of COS: the COS-STAD framework

The methodological quality of the COS development studies was evaluated by using COS-STAD recommendations, which were recently developed by the COMET initiative to improve the quality of COS development [7]. The purpose of the COS-STAD project is to identify minimum standards for the development of COS in order to strengthen the methodological approaches adopted by COS developers and to provide a framework for users to evaluate the quality of existing COS. Eleven minimum standards categorized under the three domains of scope specification, stakeholders involved and consensus process have been recommended to COS developers. For each criterion, we indicated ‘yes’ when the study fulfilled it, ‘no’ when the study did not, and ‘not applicable’ when the reported information was too limited in order to provide a judgment. By appraising the methodological quality of COS development studies, we aimed to highlight any weaknesses to bear in mind when considering COS use in clinical research or practice and to guide future COS developers by pointing out ‘gaps’ that should be addressed.

### Mapping outcomes from existing COS to RWD sources

The mapping exercise aimed at estimating the degree of overlap between the outcomes included in the COS development studies retrieved from the COMET database and the variables routinely collected in RWD sources within a European context. A coverage matrix displaying the identified outcomes in COS mapped over selected sources of RWD was produced to test the feasibility of mapping.

The relevant RWD sources were identified by: (a) examining websites, publications and descriptions of variables from European Union-funded programs (including direct research team experience with the EuroHOPE (www.eurohope.info), BridgeHealth (www.bridge-health.eu) and MedtecHTA (www.medtechta.eu*)* projects) testing the use of routinely collected administrative health data from several countries to measure outcomes; and (b) collecting information from the literature and the ClinTrials.org database regarding the type and availability of data reported in existing patient registries and patient registries linked to administrative data. The databases identified were divided into two broad categories: (1) routinely collected administrative health data at national level and (2) patient registries. Small scale observational studies and EMRs/EHRs were excluded from the mapping exercise because of considerably difficult standardization of the variables covered in these types of data sources.

1. The administrative database mapping exercise tested for COS in selected European countries (i.e., Finland, Norway, Sweden, Hungary, Italy) assuming a linked database of hospital discharge records, mortality registries, and medication purchases, which are available (with varying levels of restrictions) in many European countries (Table S1). Additional databases, including ambulatory care, primary care, long-term care, home health care, hospice, psychiatric care, and rehabilitation may be available in some countries, but tend to differ greatly in terms of level of detail, quality and completeness of the data collected and often present particular difficulties for linking data. We therefore restricted the mapping exercise to outcomes that could be reported in hospital discharge records, mortality registries, and medication purchases. An important pre-condition to outcome measurement using administrative databases is the ability to link the databases using a unique, blinded identifier for the patient, allowing for patient-level and longitudinal analyses as opposed to admission-level or service-level analyses. The methodology assumes an extraction algorithm to identify incident cases of prostate cancer, using, for example, the International Classification of Diseases, Ninth Version (ICD9) diagnosis for malignant neoplasm of the prostate (code 185) or carcinoma in situ of the prostate (code 233.4), in the primary diagnostic field or all diagnostic fields. To identify incident cases, a look-back period (usually 1 year) is applied to finalize the patient cohort, identifying an index date (*indate*) for the first appearance of the diagnosis of prostate cancer. Patient identification codes (unique and blinded) are then used to extract all follow-up care (and the one-year look-back period) in the hospital discharge database and the medication purchases database, plus the mortality register. Thus, the outcomes reported in COS development studies were mapped onto patient-level administrative data by searching for specific ICD9 diagnosis and procedure codes (the more detailed tenth version codes (ICD10) could also be used, where available), Anatomical Therapeutic Chemical (ATC) Classification System codes for medication purchases, and mortality registries for survival outcomes. The outcome was assumed measurable if at least one ICD9 (or ATC) code could be identified. For example, symptomatic outcomes related to urinary incontinence were presumed measurable by searching diagnostic code fields for the related codes (e.g., ICD9 code 788.30 - urinary incontinence, unspecified - or R32 for ICD10). Outcomes related to disease progression were assessed in relation to the ability to identify various treatments (such as surgical intervention (e.g., ICD9 procedure code 60.5 for radical prostatectomy), radiotherapy (e.g., ICD9 procedure 92.29), and chemotherapy (e.g., ICD9 procedure 99.25 or ATC L01CD02)), and/or developments of recurrences or metastases (e.g., ICD9 diagnoses 196–199), and/or mortality, all in relation to the *indate*, to map treatment trajectories and developments over time. The experience gained from several international, EU-funded projects using administrative data to measure outcomes and health care system performance informed the process through consulting methodology discussion papers and resulting publications from their websites, including EuroHOPE, BridgeHealth, and MedtecHTA. In the first two projects, administrative data were linked to form an individual-level country database to measure outcomes for specific disease areas in the same countries (i.e., Finland, Norway, Sweden, Hungary, and Italy) addressed in this study. Other EuroHOPE/BridgeHealth countries (Scotland, the Netherlands, and Denmark) did not have full access to the three linked databases and were excluded from the exercise.
2. The EMA defines patient registries as “organised systems that use observational methods to collect uniform data on a population defined by a particular disease, condition (e.g., age, pregnancy, specific patient characteristics), or exposure, and that is followed over time” [16]. The assessment of the feasibility of COS measurements using patient registries was based on a recent review which identified seven population-based and six prospective disease-specific registries currently available worldwide for prostate cancer generally, and also provided information on the outcomes measured in each registry [17]. The registry assessment was further informed by searching ClinTrials.gov (on 9 December 2019), identifying 57 active or completed studies with “prostate cancer” indicated as the “disease or condition”, and “patient registry” as the “study type”, to examine outcomes proposed for each of these studies, data sources and locations. A conservative approach was taken to select the most comprehensive patient registry in Europe based on the review [17] and ClinTrials.gov searches. The most promising candidates for COS measurement appeared to be nationally-based patient registries that were linked to administrative data. The European example chosen for full mapping of COS was the Prostate Cancer data Base Sweden (PCBaSe), which, from 2008, links the Swedish National Prostate Cancer Register (NPCR) to the Mortality Registry, the National Patient Register (hospital and outpatient care) and the prescribed drug registry, as well as other registers with patient demographic characteristics or conditions (e.g., diabetes) [18–20]. We downloaded from the NPCR website (http://npcr.se/in-english/) the forms covering diagnostics (D form), work up and treatment (Tx form), curative radiotherapy (RT form, since 2007) and radical prostatectomy (RP form, since 2015), as well as PROs (PROM form, since 2007). Variables for 5 years of follow-up information for a subset of patients (5yrf-up) were also consulted [19]. Recent expansions include the PCBaSe^Traject^ which tracks treatment trajectories for over 106,000 men through any combination of conservative treatment, radical prostatectomy, radiotherapy, androgen deprivation therapy (ADT), and gonadotropin-releasing hormone (GnRH) agonists [20], and the offshoot Patient-overview Prostate Cancer (PPC) registry for men with hormonally treated prostate cancer, especially castration-resistant prostate cancer [18].

## Results

### Data extraction: study characteristics and methodology

From a total of 19 studies retrieved from the COMET database under the ‘prostate cancer’ disease name, 1 was removed as a duplicate, 1 was unpublished, 4 were excluded because they were classified by COMET as ‘systematic reviews’ and 2 were excluded because classified as ‘recommendations’. Therefore, 11 (published between 1995 and 2017) met the inclusion criteria [21–31]; of these, 8 were classified as ‘COS for clinical trials and clinical research’, 2 as ‘COS for practice’ and 1 as ‘COS patient reported outcomes’ (Table 1). The first group of studies [21, 23–25, 27, 29–31] presented different sets of relevant endpoints to be included in future clinical trials. The two studies classified as ‘COS for practice’ developed a standard set of health outcomes, including clinical data and patient-reported outcomes, which should be measured in prostate cancer patients during routine clinical care for improving the value of treatment [26], assessing the quality of care and promoting international comparisons [28]. The only study defined as ‘COS patient reported outcomes’ [22] recommended core sets of patient-reported outcomes to be routinely incorporated in clinical trials.

**Table 1:** Characteristics of the included COS development studies in prostate cancer (*n* = 11)

First Author [ref]	Year	Study type	Target population	Methods for consensus	Type of participants	Number of participants	Geographical origin
Auvinen A [21]	1996	COS for clinical trials or clinical research	Men aged 56–67 eligible for screening	Meeting	Clinical experts; non-clinical research experts	26	Europe, Canada, United States
Chen RC [22]	2014	COS patient reported outcomes	Adult patients (19+) with localized and advanced cancer	Systematic literature review; meeting	Clinical experts; non-clinical research experts; public representatives	NA	NA
Dawson NA [23]	1998	COS for clinical trials or clinical research	Patients with hormone-refractory prostate cancer	Survey	Clinical experts	35	Europe, Canada, United States
Denis L [24]	1997	COS for clinical trials or clinical research	Especially patients with localized cancer	Meeting	Clinical experts; others	6	Europe, United States
MacLennan S [25]	2017	COS for clinical trials or clinical research	Patients aged 45–75 with localized prostate cancer undergoing surgery, active surveillance, ablative therapy, hormonal therapy	Meeting; Delphi; focus group; systematic literature review	Clinical experts; public representatives	105 patients + 47 clinical experts	Europe, United States
Martin NE [26]	2015	COS for practice	Men with newly diagnosed localized (stages T1-T4) cancer treated with curative intent or followed-up with active surveillance	Delphi; teleconferences; survey	Clinical experts; non-clinical research experts; public representatives	NA	NA
Middleton RG [27]	1995	COS for clinical trials or clinical research	Patients with stage T2(B) prostate cancer undergoing radical prostectomy, radiation therapy, or surveillance	Literature review	NA	NA	United States
Morgans A [28]	2015	COS for practice	Metastatic and recurrent patients ineligible for further curative therapy	Delphi; literature review; teleconferences	Clinical experts; non-clinical research experts; public representatives	25	Europe, Australia, Canada, United States
Schellhammer P [29]	1997	COS for clinical trials or clinical research	Patients with localized cancer	Literature review; conference	Clinical experts; others	NA	Europe, Canada, United States
van den Bos W [30]	2014	COS for clinical trials or clinical research	Patients with localized cancer and candidates for focal therapy	Delphi; meeting; literature review	Clinical experts	48	North America, Europe and Asia
van den Bos W [31]	2015	COS for clinical trials or clinical research	Patients with localized recurrent disease after radiation therapy	Delphi; meeting; literature review	Clinical experts	55	NA

*COS* Core outcome set, *NA* Not available

The types of participants involved in COS development were classified based on the list suggested by Gargon [13] and included seven possibilities: clinical experts, public representatives, non-clinical research experts, authorities, industry representatives, others (e.g., ethicists), or no details given. Each study could involve multiple participant categories. ‘Clinical experts’ was the most prevalent category of participants (91% of studies), followed by ‘non-clinical research experts’ (e.g., epidemiologists and health economists; 36%), ‘public representatives’ (e.g., patients, caregivers and patient associations; 36%), and ‘others’ (18%); no details were reported in 9% of the studies.

Overall, 73% of studies recruited COS development participants from North America and 64% from Europe; conversely, other continents such as Asia and Australia were weakly represented in COS development (9% each). In details, one study [27] recruited participants from the US only, two [24, 25] from Europe and US, three [21, 23, 29] from Europe, US and Canada, one [30] from North America, Europe and Asia, and one [28] from Europe, US, Canada and Australia. No information on participant locations were given in three studies [22, 26, 31]. The number of participants was reported in 7 studies only and ranged between 6 [24] and 152 [25], with a median of 35 cross the studies.

The methods used to develop consensus were classified as reported in the COMET database. Each study may have used multiple methodologies. The techniques adopted were heterogeneous and included: systematic literature reviews (64%), consensus meetings (54%), Delphi technique (45%), surveys (18%), teleconferences (18%), consensus conferences (9%) and focus groups (9%).

### Data extraction: outcome classification, outcomes, and outcome measurement instruments (OMIs)

All the 11 studies retrieved reported information of “what” to measure in terms of outcomes. Based on the outcome taxonomy adopted [15], eight studies recommended outcomes belonging to ‘mortality/survival’ domain, seven addressed the ‘outcomes related to neoplasms’ and six the ‘renal and urinary outcomes’. Studies [22, 26, 28] classified as ‘COS for practice’ and ‘COS patient reported outcomes’ were more likely to recommend outcomes belonging to ‘renal and urinary outcomes’, ‘gastrointestinal outcomes’, ‘endocrine outcomes’, ‘reproductive system outcomes’, and ‘general outcomes’ (e.g., pain) that, indeed, are generally self-reported by patients. Conversely, the bulk of studies classified as ‘COS for clinical trials or clinical research’ were more prone to address the ‘outcomes related to neoplasms’ domain, which generally requires a clinical assessment by the physician. Obviously, the ‘mortality/survival’ domain was not applicable to the study classified as ‘COS patient reported outcomes’ [22].

**Fig. 1:**
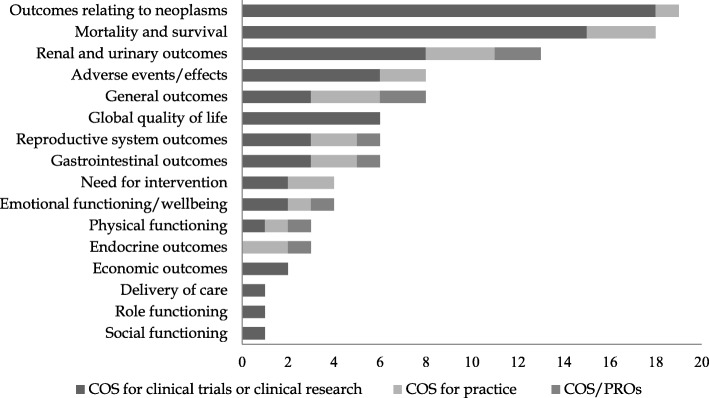
Number of outcomes included in COS according to outcome category and study type

Moreover, we identified an average of 9 outcomes per study reported and 103 outcomes listed in total. As expected from the study-level analysis, most of them were classified into ‘outcomes related to neoplasms’ (18%), ‘mortality/survival’ (17%), and ‘renal and urinary outcomes’ (13%) categories; other classes including more than 5% of the outcomes were ‘general outcomes’, ‘adverse events’, ‘gastrointestinal outcomes’, ‘reproductive system outcomes’, and ‘global quality of life’. In terms of single outcomes, (overall and cause-specific) survival and quality of life were the most frequently reported by studies. A synthetic representation of outcome categories according to study type is displayed in Fig. 1; full details of outcome categories, together with the number of outcomes and studies belonging to each of them, are shown in Table 2.

**Table 2:** List of outcome categories/outcomes in COS from the included studies by study type

Outcome classification/Outcomes	All COS studies (*n* = 11);		COS for clinical trials or clinical research (*n* = 8)		COS for practice (*n* = 2)		COS/patient reported outcomes (*n* = 1)	
	No. O	No. S	No. O	No. S	No. O	No. S	No. O	No. S
*Mortality and survival*	*18*	*8*	*15*	*6*	*3*	*2*	*NA*	*NA*
Survival	2		2		0		NA	
Overall survival	5		3		2		NA	
Cause- (or disease) specific survival	6		5		1		NA	
Relative survival	1		1		0		NA	
Metastasis-free survival	2		2		0		NA	
Progression-free survival	1		1		0		NA	
Biochemical recurrence-free survival	1		1		0		NA	
*Outcomes relating to neoplasms*	*19*	*7*	*18*	*6*	*1*	*1*	*0*	*0*
(Change in) prostate-specific antigen (PSA) levels	3		3		0		0	
Measurable disease response	1		1		0		0	
Time to progression	2		2		0		0	
Disease progression/Progression rate	2		2		0		0	
Progression-free probability	1		1		0		0	
Development of metastases	1		1		0		0	
Metastases-free probability	1		1		0		0	
Symptomatic skeletal event	1		0		1		0	
Local disease	1		1		0		0	
Positive surgical margins	1		1		0		0	
Response duration	1		1		0		0	
Failure-free probability	1		1		0		0	
Development of castration-resistant disease	1		1		0		0	
Treatment failure	2		2		0		0	
*Renal and urinary outcomes*	*13*	*6*	*8*	*3*	*3*	*2*	*2*	*1*
Urinary incontinence	4		2		1		1	
Urinary obstruction/irritation	3		1		1		1	
Urinary symptoms	1		0		1		0	
Voiding behaviour	1		1		0		0	
Haematuria	1		1		0		0	
Pelvic pain	1		1		0		0	
Lymphedema	1		1		0		0	
Urinary functioning	1		1		0		0	
*Gastrointestinal outcomes*	*6*	*5*	*3*	*2*	*2*	*2*	*1*	*1*
Bowel symptoms	3		0		2		1	
Faecal incontinence	1		1		0		0	
Bowel functioning	1		1		0		0	
Diarrhoea	1		1		0		0	
*Endocrine outcomes*	*3*	*3*	*0*	*0*	*2*	*2*	*1*	*1*
Hormonal symptoms	3		0		2		1	
*Reproductive system outcomes*	*6*	*6*	*3*	*3*	*2*	*2*	*1*	*1*
Erectile/sexual function	2		2		0		0	
Erectile/sexual dysfunction (impotence)	3		1		1		1	
Sexual symptoms	1		0		1		0	
*General outcomes*	*8*	*3*	*3*	*1*	*3*	*1*	*2*	*1*
Pain	2		0		1		1	
Fatigue	2		0		1		1	
Bone pain	1		1		0		0	
Weight loss	1		1		0		0	
Anaemia	1		1		0		0	
Performance status	1		0		1		0	
*Physical functioning*	*3*	*3*	*1*	*1*	*1*	*1*	*1*	*1*
Physical wellbeing/functioning	3		1		1		1	
*Emotional functioning/wellbeing*	*4*	*4*	*2*	*2*	*1*	*1*	*1*	*1*
Mental/emotional wellbeing/functioning	4		2		1		1	
*Social functioning*	*1*	*1*	*1*	*1*	*0*	*0*	*0*	*0*
Social functioning	1		1		0		0	
*Role functioning*	*1*	*1*	*1*	*1*	*0*	*0*	*0*	*0*
Role functioning	1		1		0		0	
*Global quality of life*	*6*	*6*	*6*	*6*	*0*	*0*	*0*	*0*
Quality of life	6		6		0		0	
*Economic outcomes*	*2*	*2*	*2*	*2*	*0*	*0*	*0*	*0*
Cost-effectiveness	1		1		0		0	
Costs	1		1		0		0	
*Need for intervention*	*4*	*2*	*2*	*1*	*2*	*1*	*0*	*0*
Need for salvage therapy	1		1		0		0	
Need for curative treatment	1		1		0		0	
Need for pain medication	1		0		1		0	
Procedures need for local progression	1		0		1		0	
*Delivery of care*	*1*	*1*	*1*	*1*	*0*	*0*	*0*	*0*
Time to treatment failure	1		1		0		0	
*Adverse events/effects*	*8*	*6*	*6*	*4*	*2*	*2*	*0*	*0*
Adverse events	3		2		1		0	
Perioperative deaths	1		1		0		0	
Thromboembolic disease	1		1		0		0	
Bothersome or symptomatic urethral or anastomotic stricture	1		1		0		0	
Side-effects of hormonal therapy	1		1		0		0	
Major systemic therapy effects	1		0		1		0	
Total	**103**		**72**		**22**		**9**	

*NA* Not applicable,

Additionally, seven COS development studies [21, 22, 26, 28–31] reported details on “how” to measure the outcomes proposed (i.e., the recommended OMIs, outcome measurement timing and outcome measurement sources), making it possible to classify data sources for outcome measurement into the following classes: (1) clinical data (e.g., prostate-specific antigen levels; 4 studies); (2) administrative data (i.e., death certificate; 3 studies); (3) PROMs (e.g., Expanded Prostate Cancer Index Composite (EPIC-26); 7 studies). Multiple classes of outcome measurement sources were possible for each study. PROMs were recommended more often in the three studies [22, 26, 28] classified as ‘COS for practice’ or ‘COS patient reported outcomes’ and involving public representatives in the COS development process. In these studies, indeed, at least six different outcome categories were recommended to be patient-reported, compared to less than three in the studies classified as ‘COS for clinical trial or clinical research’ [21, 29–31].

### Assessing the quality of COS through the COS-STAD framework

Table 3 presents the COS-STAD review for the COS development studies retrieved in prostate cancer. All 11 studies identified the setting, health condition and population covered by the COS; eight studies specified the intervention covered. In the stakeholder involvement domain, three out of the 11 studies included those who will use COS in research; seven studies included healthcare professionals experienced in treating patients with the condition, but only four included patients or their representatives. In the consensus process domain, five out of the 11 studies considered both healthcare professionals’ and patients’ views in drafting the initial list of outcomes; seven studies specified the scoring process a priori, and six specified the criteria for including or dropping outcomes a priori. One study only specified the measures taken to avoid ambiguity of language used in the outcomes list. Overall, the number of recommendations addressed (i.e., coded as ‘yes’ in the table) by each study ranged between three [24] and ten [25], averaging at 5.7 across the studies and increasing in more recently published ones. The three best performing papers identified were those by MacLennan [25] for the ‘COS for clinical trials or clinical research’ group, and by Martin [26] and Morgans [28] within the ‘COS for practice’ one.

**Table 3:** COS-STAD review for the included COS development studies

	Study	Auvinen 1996 [21]	Chen 2014 [22]	Dawson 1998 [23]	Denis 1997 [24]	MacLennan 2017 [25]	Martin 2015 [26]
	Study type	COS for clinical trials or clinical research	COS patient reported outcomes	COS for clinical trials or clinical research	COS for clinical trials or clinical research	COS for clinical trials or clinical research	COS for practice
	Methods for consensus	Semi structured discussion; meeting	Systematic literature review; consensus meeting	Survey	Semi structured discussion: meeting	Consensus meeting; Delphi; focus groups; systematic review	Modified Delphi; teleconference; surveys
Domain	Minimum standard						
Scope specification	The research or practice setting(s) in which the COS is to be applied	Yes	Yes	Yes	Yes	Yes	Yes
	The health condition(s) covered by the COS	Yes	Yes	Yes	Yes	Yes	Yes
	The population(s) covered by the COS	Yes	Yes	Yes	Yes	Yes	Yes
	The intervention(s) covered by the COS	Yes	Yes	NR	NR	Yes	Yes
Stakeholders involved	Those who will use the COS in research	Yes	NR	Yes	NR	NR	No
	Healthcare professionals with experience of patients with the condition	NR	Yes	Yes	NR	Yes	Yes
	Patients with the condition or their representatives	No	Yes	No	NR	Yes	Yes
Consensus process	Initial list of outcomes considered both healthcare professionals’ and patients’ views	NR	No	Yes	NR	Yes	Yes
	A scoring process and consensus definition were described a priori	NR	NR	NR	NR	Yes	NR
	Criteria for including/dropping/adding outcomes were described a priori	NR	NR	NR	NR	Yes	NR
	Care was taken to avoid ambiguity of language used in the list of outcomes	NR	No	NR	NR	Yes	NR
	Study	Middleton 1995 [27]	Morgans 2015 [28]	Schellhammer 1997 [29]	Van den Bos 2014 [30]	Van den Bos 2015 [31]	
	Study type	COS for clinical trials or clinical research	COS for practice	COS for clinical trials or clinical research	COS for clinical trials or clinical research	COS for clinical trials or clinical research	
	Methods for consensus	Literature review	Modified Delphi; literature review; teleconferences	Literature review; conference	Modified Delphi; consensus meeting; literature review	Delphi process; consensus meeting; literature review	
Domain	Minimum standard						
Scope specification	The research or practice setting(s) in which the COS is to be applied	Yes	Yes	Yes	Yes	Yes	
	The health condition(s) covered by the COS	Yes	Yes	Yes	Yes	Yes	
	The population(s) covered by the COS	Yes	Yes	Yes	Yes	Yes	
	The intervention(s) covered by the COS	Yes	Yes	NR	Yes	Yes	
Stakeholders involved	Those who will use the COS in research	NR	No	NR	Yes	NR	
	Healthcare professionals with experience of patients with the condition	NR	Yes	NR	Yes	Yes	
	Patients with the condition or their representatives	No	Yes	NR	No	No	
Consensus process	Initial list of outcomes considered both healthcare professionals’ and patients’ views	No	Yes	Yes	No	No	
	A scoring process and consensus definition were described a priori	NR	NR	NR	NR	NR	
	Criteria for including/dropping/adding outcomes were described a priori	NR	NR	NR	NR	NR	
	Care was taken to avoid ambiguity of language used in the list of outcomes	No	NR	NR	No	NR	

*COS* Core outcome set, *COS-STAD* COS-STAndards for Development, *NR* Nothing reported

### Mapping outcomes from existing COS to RWD sources

The mapping exercise explored the potential to measure the outcomes included in the COS development studies by using RWD. Table 4 illustrates the mapping exercise results for all the outcomes identified in Table 2, highlighting those from the three highest quality studies [25, 26, 28] based on the COS-STAD review; moreover, two [26, 28] of these were classified as ‘COS for practice’, allowing an interpretation of the mapping results also based on the type of study.

**Table 4:** Mapping COS onto RWD sources in selected European countries: an exercise in feasibility

Outcome classification/Outcomes			Linked administrative databases (Finland, Norway, Sweden, Hungary, Italy)				Prostate Cancer data Base Sweden (PCBaSe): National Prostate Cancer Register (NPCR) linked to administrative databases (Sweden)	
High-quality study	COS for practice		Hospital discharges (H)	Mortality (M)	Purchased medication (PM)	Notes		Notes
		*Mortality and survival*						
		Survival	√	√		Requires identifying patients in H and measuring mortality in M	√	NPCR D plus Mortality PCBaSe
√	√	Overall survival	√	√		Requires identifying patients in H and measuring mortality in M	√	NPCR D plus Mortality; PCBaSe
√	√	Cause (disease) specific survival	√	√		Requires identifying patients in H and measuring cause of death in M	√	NPCR D plus Mortality; PCBaSe
		Relative survival	√	√		Requires comparing the above measures to a similar population (by age, sex) without the disease	√	NPCR D plus Mortality; PCBaSe
√	√	Metastasis-free survival	√	√		Requires measuring survival in patients for whom no metastatic carcinoma codes (codes 196–199) or secondary cancers are discernible over time	√	NPCR 5yrf-up; PCBaSe
		Progression-free survival	√	√		Requires stratifying patients for evidence of progression compared to those without, using codes identified as indicating disease progression (e.g., castration-resistant disease, metastases, recurrence)	√	NPCR 5yrf-up; NCPR Tx; PCBaSe
√	√	Biochemical recurrence-free survival				No clinical test data is available	√	NPCR 5yrf-up includes PSA levels at diagnosis and over time (changes), some indications in NPCR RT or RP
		*Outcomes relating to neoplasms*						
		(Change in) prostate-specific antigen (PSA) levels				Clinical test data are not available but the code for elevated prostate specific antigen (PSA) (e.g., 790.93) could be checked over time in the H	√	NPCR 5yrf-up includes PSA levels at diagnosis and over time (changes), some indications in NPCR RT or RP
		Measurable disease response				Clinical data are not available		Might be possible using NPCR, but only RT and RP forms collect imaging data
		Time to progression	√			Requires identifying codes for indicating progression (e.g., castrationresistant disease, metastases, recurrence)	√	NPCR 5yrf-up; NPCR Tx; PCBaSe
√		Disease progression/Progression rate	√			Requires identifying codes for indicating progression (e.g., castrationresistant disease, metastases, recurrence)	√	NPCR 5yrf-up; NPCR Tx; PCBaSe
		Progression-free probability	√			Requires measuring probability using the outcome above	√	NPCR 5yrf-up; PCBaSe
√	√	Development of metastases	√			Requires identifying codes for metastatic carcinoma (196–199) or secondary cancers	√	NPCR 5yrf-up; PCBaSe
		Metastases-free probability	√			Requires measuring probability using the outcome above	√	NPCR 5yrf-up; PCBaSe
√	√	Symptomatic skeletal event	√			Requires identifying codes for pathologic fracture (e.g., 733.1), surgery or radiation to bone (e.g., ICD9 procedure codes indicating bone surgery, 79*) or radiation (e.g., ICD9 V58.0) combined with bone diagnoses, or spinal cord compression (e.g., 336.9)	√	NPCR 5yrf-up; PCBaSe
√		Local disease				Local disease recurrence would be difficult to discern, except the case where patients with established diagnoses (e.g., 185) over time, present later with an in situ diagnosis (233.4)		Difficult to discern but could follow technique at left; also may be helpful to stratify patients according to NPCR Tx.
√		Positive surgical margins					√	NPCR 5yrf-up; NPCR RP
		Response duration						
		Failure-free probability						
		Development of castration-resistant disease^20^	√		√	Requires identifying patients with evidence of surgical castration (ICD9 procedure codes 62.3, 62.41, 62.42) or medical castration using ADT (e.g., ATC code L02BB03), noting also abiraterone (ATC L02BX03), and disease progression (e.g., metastases codes 196–199) subsequent to ADT or abiraterone; check also for elevated PSA (e.g., 790.93).	√	NPCR 5yrf-up; NPCR RP subgroup; PCBaSe
√		Treatment failure	√	√	√	Requires measuring mortality and/or evidence of disease progression (e.g., metastases) for groups of patients stratified for various treatments (e.g., radical prostatectomy, ADT)	√	NPCR 5yrf-up for failure of conservative therapy; PCBaSe
		*Renal and urinary outcomes*						
√	√	Urinary incontinence	√			Requires identifying relevant codes (e.g., 788.30)	√	NPCR PROMs; PCBaSe
√	√	Urinary obstruction/irritation	√			Requires identifying relevant codes (e.g., 599.60)	√	NPCR PROMs; PCBaSe
√	√	Urinary symptoms	√			Requires identifying relevant codes (e.g., 788*)	√	NPCR PROMs; NPCR 5yrf-up; PCBaSe
		Voiding behaviour	√			Requires identifying relevant codes (e.g., 596.59, 788.69)	√	NPCR PROMs; NPCR 5yrf-up; PCBaSe
		Haematuria	√			Requires identifying relevant codes (e.g., 599.7)	√	PCBaSe
		Pelvic pain	√			Requires identifying relevant codes (e.g., 608.9)	√	PCBaSe
		Lymphedema	√			Requires identifying relevant codes (e.g., 457.1)	√	PCBaSe
√		Urinary functioning	√			Requires identifying relevant codes (e.g., 788*)	√	NPCR PROMs; NPCR 5yrf-up; PCBaSe
		*Gastrointestinal outcomes*						
√	√	Bowel symptoms	√			Requires identifying relevant codes (e.g., 787.99)	√	NPCR PROMs; NPCR 5yrf-up; PCBaSe
√		Faecal incontinence	√			Requires identifying relevant codes (e.g., 787.6)	√	NPCR PROMs; PCBaSe
√		Bowel functioning	√			Requires identifying relevant codes (e.g., 787.99)	√	NPCR PROMs; NPCR 5yrf-up; PCBaSe
		Diarrhoea	√			Requires identifying relevant codes (e.g., 787.91, 564.5)	√	PCBaSe
		*Endocrine outcomes*						
√	√	Hormonal symptoms	√		√	Requires identifying relevant codes related to side-effects from hormonal treatments (e.g., fatigue 780.79, weight loss 783.21) in patients with evidence of ADT (e.g., ATC code L02BB03), checking for chemotherapy with docetaxel (ATC code L01CD02) in PM.	√	NPCR 5yrf-up; NPCR Tx; PCBaSe
		*Reproductive system outcomes*						
√		Erectile/sexual function	√			Requires identifying relevant codes (e.g., 607.84 and/or procedure codes 60.94, 60.95, 60.96, 60.97)	√	NPCR PROMs; PCBaSe
√	√	Erectile/sexual dysfunction (impotence)	√			Requires identifying relevant codes (e.g., 607.84 and/or procedure codes 60.94, 60.95, 60.96, 60.97)	√	NPCR PROMs; PCBaSe
√	√	Sexual symptoms	√			Requires identifying relevant codes (e.g., 607.84 and/or procedure codes 60.94, 60.95, 60.96, 60.97)	√	NPCR PROMs; PCBaSe
		*General outcomes*						
√	√	Pain	√		√	Requires identifying relevant codes (e.g., 338*, 780.96, various) in H, ATC codes (N02*) in PM	√	PCBaSe
√	√	Fatigue	√			Requires identifying relevant codes (e.g., 780.79)	√	PCBaSe
		Weight loss	√			Requires identifying relevant codes (e.g., 783.21)	√	PCBaSe
		Bone pain	√		√	Requires identifying relevant codes (e.g., 733.90), though the code is not specific only to bone pain, suggesting a need for further identification of codes for pain (e.g., 388*) in H and ATC codes for pain medications (e.g., N02*) in PM	√	PCBaSe
		Anaemia	√			Requires identifying relevant codes (e.g., 285.9)	√	PCBaSe
√	√	Performance status						
		*Physical functioning*						
√	√	Physical wellbeing/functioning					√	NPCR PROMs
		*Emotional functioning/wellbeing*						
√	√	Mental/emotional wellbeing/functioning				Not possible, although some measures of well-being, such as depression codes (e.g., 311, 2962 or 2963) in H or anti-depressives (ATC code N06A) in PM could be identified.	√	NPCR PROMs
		*Social functioning*						
		Social functioning						
		*Role functioning*						
		Role functioning						
		*Global quality of life*						
√		Quality of life					√	NPCR PROMs
		*Economic outcomes*						
		Cost-effectiveness	√		√	Requires measuring costs in H, e.g., using DRG tariffs, and medication costs in PM, and constructing cost-effectiveness measures for various treatments and outcomes	√	PCBaSe
		Costs	√		√	Requires measuring costs in H, e.g., using DRG tariffs, and medication costs in PM	√	PCBaSe
		*Need for intervention*						
√		Need for salvage therapy				Salvage therapies might be partially identified by stratifying patients for therapies, e.g., radical prostatectomy (60.5 procedure code) followed by external beam radiotherapy (92.29 procedure code) or ADT (e.g., ATC L02BB03)	√	NPCR 5yrf-up, to some extent; NPCR Tx; PCBaSe, to some extent
√		Need for curative treatment				This outcome seems to refer specifically to patients under active surveillance, difficult to ascertain, other than by observing a total lack of treatment upon and after diagnosis, followed by eventual treatment after some time.	√	NPCR 5yrf-up, related to questions regarding reasons for terminating conservative therapy
√	√	Need for pain medication	√		√	Requires identifying relevant codes for pain (e.g., 338*, 780.96) in H, or pain medication (ATC codes N02*) in PM	√	PCBaSe
√	√	Procedures need for local progression				As described for “need for salvage therapy” or “local disease”, such measures could follow a similar logic.		PCBaSe, to some extent
		*Delivery of care*						
		Time to treatment failure						
		*Adverse events/effects*						
√	√	Adverse events	√		√	Requires stratifying patients by therapy and then measuring any adverse events, i.e., complications of surgical and medical care, not elsewhere classified (996-999), urinary complications (997.5), bowel obstruction (560.9) or effects of radiation, unspecified (990). See also “major systemic therapy effects”.	√	NPCR 5yrf-up; NPCR Tx; NPCR RP; NPCR RT; NPCR PROMs; PCBaSe
√		Perioperative deaths	√	√		Requires measuring in-hospital deaths for surgical admissions and deaths within a certain timeframe from surgery (M) to give a (potentially incomplete) indication of this outcome	√	PCBaSe
√		Thromboembolic disease	√			Requires identifying relevant codes (e.g., 451*, 453*), pulmonary embolism (415.1), peripheral arterial occlusion disease (443.9), deep vein thrombosis codes, especially for ADT patients. Possibly also related procedure codes (e.g., venography, 886*).	√	PCBaSe
√		Bothersome or symptomatic urethral or anastomotic stricture	√			Requires identifying relevant codes (e.g., 598.9, 997.49).	√	NPCR 5yrf-up; NPCR PROMs; PCBaSe
√		Side-effects of hormonal therapy	√		√	Requires identifying relevant codes related to side-effects from hormonal treatments (e.g., fatigue 780.79, weight loss 783.21) in ADT patients (e.g., ATC code L02BB03). Also, see “Hormonal symptoms”.	√	NPCR 5yrf-up; NPCR Tx; PCBaSe
√	√	Major systemic therapy effects	√		√	Requires stratifying patients into groups with evidence of systemic therapy, i.e., hormonal therapy (e.g., ATC L02BB04 in PM), chemotherapy (e.g., docetaxel L01CD02 or cabazitaxel L01CD04 in PM), immunotherapy (e.g., sipuleucel-T L03AX17), treatments for bone metastases (e.g., denosumab M05BX53 in PM). Evidence of side effects are then sought in H and/or PM.	√	NPCR 5yrf-up; NPCR Tx; PCBaSe

LEGEND: √ indicates that the outcome comes from a high-quality study [25, 26, 28] or from a 'COS for practice' one [26, 28], and that measures for the outcome can be constructed using the source of the data. *Blank*: There is no evidence of information in the database(s) that can be used to measure the outcome

A description of the country-level databases investigated for the mapping exercise is provided in Table S1. All codes refer to (ICD9) diagnostic codes unless otherwise indicated. ATC codes refer to medications. All techniques assume that time is measured from incidence (first date of diagnosis, *indate*) to evidence of the code(s) for the symptom, treatment or outcome. In PCBaSe data, NPCR 5yrf-up are variables collected to measure 5-year follow-up for a group of patients with incident prostate cancer registered between 2003 and 2005 [19]. We assume administrative database techniques are used with the PCBaSe, including its recent developments (i.e. PCBaSe^Traject^ tracking treatment trajectories over time, and Patient-overview Prostate Cancer (PPC) for hormonally treated prostate cancer) [18, 20]

*Abbreviations*: *ICD9* International Classification of Diseases (Ninth Edition), *ATC* Anatomical Therapeutic Chemical Classification System, *PSA* Prostate-specific antigen, *ADT* Androgen deprivation therapy, *DRG* Diagnosis-Related Group. *NPCR D* NPCR diagnostic (form), *NPCR Tx* NPCR work up and treatment (form), *NPCR RT* NPCR curative radiotherapy (form), *NPCR RP* NPCR radical prostatectomy (form), *NPCR PROMs* NPCR PROMs (form)

Using linked, patient-level administrative data over time, we showed how diagnostic and procedure codes (ICD9, ICD10 and/or country-specific procedure coding systems), ATC codes for medications, mortality dates and causes could be used to measure COS. All outcomes for mortality and survival are measurable, with the exception of biochemical recurrence-free survival, which requires clinical measures unavailable in administrative data. There may be difficulty in establishing a definitive first diagnosis date, or index date (*indate*), and information on staging, grade, initial prostate-specific antigen (PSA) level (and changes in PSA levels) and risk categories for patients are not available, nor are PROMs. Progression-free survival is measurable, but would require clear assumptions for codes to identify evidence of progression, and depends on the reliability of the coding and completeness of the data over a sufficient period of time. Outcomes relating to neoplasms that require clinical data (e.g., PSA level changes, measurable disease, local disease, positive surgical margins) are not measurable, while those related to progression are measurable insofar as patients can be stratified into various, recognizable treatment and outcome trajectories. Codes were identified for renal and urinary, gastrointestinal, endocrine, reproductive system and general outcomes (with the exception of performance status), and so can be searched and measured in relation to the *indate*. Adverse events/effects outcomes are measurable in ways similar to progression and symptomatic outcomes. Functioning outcomes, which rely largely on PROs, and need for intervention outcomes are not readily measurable with administrative data.

All outcomes identified from the COS development studies that are measurable using linked administrative databases can also be measured using the Swedish PCBaSE (NPCR patient registry for prostate cancer linked to administrative data), by applying the same techniques. In contrast to linked administrative data alone, patient registry information allows for definitive identification of incidence (*indate*) for all patients as well as stratification of patients according to diagnostic criteria to indicate tumour stage, grade (Gleason), risk level and initial PSA levels, initial treatment, and information for radical prostatectomy and radiotherapy as well as PROs. In Table 4, we mapped the various forms and follow-up variables identified for use in measuring the outcomes. In NPCR, the radical prostatectomy form expands the measurement of COS to include surgical margins and, along with the RT form, follow-up clinical measures like PSA level changes and thus some evidence of biochemical recurrence. The 5yrf-up patient group variables were the most useful for identifying the COS, especially for disease progression, need for intervention and adverse effects. The PROM form adds quality of life, and physical and emotional functioning to the measurable COS list, along with questions on urinary, gastrointestinal and sexual outcomes to supplement measurement using the PCBaSe. The PCBaSe, particularly the PCBaSe^Traject^ and PPC expansions, is instrumental in measuring almost the entire COS identified. We could not find sufficient information to assume feasible measurement of the following outcomes: measurable disease response, local disease, procedures need for local progression, response duration, time to treatment failure, failure-free probability, performance status, social functioning, role functioning.

Outcomes recommended by high quality studies [25, 26, 28] presented good coverage in the examined RWD sources, with no relevant differences according to the study type (i.e., ‘COS for clinical trials and clinical research [25] versus ‘COS for practice’ [26, 28]), nor to the cancer stage addressed (i.e., localized cancer [25, 26] versus advanced cancer [28]).

## Discussion

### Synthesis of results

This research aimed at identifying COS development studies in prostate cancer, critically appraising their methodological quality, and exploring the extent to which recommended COS are measurable in available RWD sources through a mapping exercise. Using a scoping review approach, the COMET database was searched in order to identify the relevant COS development studies. A total of 11 studies were finally included in the analyses. Most of the studies are classified as ‘COS for clinical trials and clinical research’, few of them are classified as ‘COS for practice’ or ‘COS patient reported outcome’, and this difference was considered in analysing study methodology and findings. Overall, few studies reported details on how recommended outcomes should be measured, including information on recommended OMIs, outcome measurement timing and sources; more recent studies provided more information of this type. Moreover, some of the retrieved studies were published more than 10 years ago and therefore outdated with respect to current clinical practice and technological opportunities (although the COMET database is updated annually and revisions to existing COS would have been captured through this update). No relevant differences were observed in terms of OMIs between COS development studies according to study type; however, the few studies classified as ‘COS for practice’ or ‘COS patient reported outcome’ were more likely to recommend the use of PROMs for outcome measurement.

The study quality assessment using the COS-STAD framework identified several limitations in the methods used to develop COS in prostate cancer. The recommended standards within the ‘scope specification’ domain were followed by most studies but there were notable gaps in properly reporting the ‘stakeholders involved’ and ‘consensus process’ adopted. In particular, patients’ involvement in the COS development process was found to be insufficient, with only four (out of eleven) studies including or reporting them. In addition, geographic representativeness of stakeholders was unbalanced in favour of Europe and North America, with lower involvement of stakeholders from other continents. Similarly, the recommended standards within the consensus process domain were poorly tracked (or reported) across the studies. Not all recommended outcomes are patient-relevant outcomes, some are biomarkers (e.g., PSA level) or so called intermediate outcomes (e.g., time to progression). When these biomarkers or intermediate outcomes are used to assess the effectiveness of an intervention (instead of, for instance, establishing a diagnosis) they are used as surrogate outcomes, that is as a replacement for a patient-relevant outcome. Surrogate endpoints should not be used unless the validity of the relation between the surrogate and the final outcome has been established in advance by means of adequate epidemiological and statistical analyses [32]. This recommendation may be added as one of the methodological criteria to establish the quality of COS studies that include putative surrogate outcomes.

Future COS users should make note of these limitations if they plan to adopt these COS; for example, whereas a COS was developed without the involvement of patients or their representatives, then the final COS is unlikely to reflect their views. The same considerations apply to any other key stakeholder group excluded from the COS development process, such as researchers. Moreover, shortcomings within the consensus process domain increase the likelihood of introducing bias into the COS; for example, if the scoring process and definition of consensus is not specified a priori, then the COS developers might change the criteria after obtaining results from a Delphi survey. Therefore, future COS users are recommended to critically evaluate a COS a priori using the COS-STAD framework to identify any potential limitations; in some cases, a new COS must be developed to address these gaps. It would also be relevant for authors and researchers involved in COS development to follow published guidelines to improve reporting of this type of studies, such as the newly developed COS-STAndards for Reporting (COS-STAR) [33]. In some circumstances, indeed, the methodological quality might erroneously appear lower because of poor reporting from the studies.

In the ‘mapping’ exercise, we estimated a 72% (44/61 outcomes covered) amount of overlap between COS reported in high-quality studies and linked administrative databases, with at least nine more (52/61, or 85%) using patient registry linked to administrative data sources. Attesting to this, many recent studies, including for prostate cancer, have applied a disease-based approach using these types of data to measure outcomes, enhanced by statistical methodologies to address selection bias, confounding and adjust for risk [17–20, 34–36]. Nevertheless, our conclusions regarding COS measurement feasibility may be overly positive; a recent study investigating the feasibility of replicating clinical trials published in high-impact journals using observational administrative or EHR data in the US found only 15% overlap [37]. The study covered interventional studies for various diseases published over 1 year and found considerable problems, especially related to clinical endpoints. We also observed such weaknesses in administrative and registry data here, particularly where the outcomes measured treatment response. However, the nature of prostate cancer as an increasingly long-term, chronic disease arguably better lends itself to measures over time using larger databases. The performed exercise suggests that treatment trajectories can be mapped to stratify patients and compare survival outcomes, as well as search for evidence of symptoms, treatment effects (i.e., incontinence, sexual dysfunction), metastases and complications. However, using predefined algorithms to measure metastases and secondary tumours in administrative data alone has proven challenging elsewhere and should therefore be tested [38]. The reliability and completeness of coding for symptoms and treatment effects in administrative data can be sub-optimal, given marked variability in number and detail of diagnostic and procedure codes in administrative data, as has been found in multi-country projects [39–41]. Specific codes may be identified for some outcomes, but whether they are routinely recorded in hospital discharge summaries needs evaluation. The ability to follow large cohorts of patients over time using administrative databases, nonetheless, can provide useful information regarding patient comorbidities and resource use, mortality outcomes, and permit large-scale comparisons of geographic areas, including cross-country [35, 39–41]. On the other end, PROMs, some clinical data (i.e., test results for treatment response, surgical margins) and functioning outcomes are lacking when considering administrative data alone. These gaps reflect the nature of these databases that were initiated for administrative purposes and are particularly intended for measuring resource use.

Linking administrative data to patient registry data allows for considerable improvement in determining incidence and matching treatments found in the administrative data with patient groups according to tumour type and risk level, though not all cancer registries contain reliable codes or geographic coverage, as was found in a EuroHOPE study for breast cancer [41]. In that study, countrywide cancer registry data was impossible to obtain for linkage in Italy, and staging information was largely incomplete. The plethora of studies available for established registry programs linked to administrative data for prostate cancer in Sweden (PCBaSe), Norway (Prostate Cancer Clinical Registry) and the US (the Surveillance, Epidemiology, and End Results (SEER) database, linked with Medicare claims data, SEER-Medicare), however, show the potential of these databases for use in population-based, observational studies. Such studies, indeed, allow for the measurement of survival and disease progression, various treatment comparisons and effects, PROMs and costs, and can provide important information regarding follow-up and comorbidities [17–20, 34, 36, 42]. In our Swedish example, the accuracy and completeness of measurement of most COS in comparison to administrative data alone is enhanced by specific reporting requirements in the forms. But coverage may still be an issue. For example, information related to primary diagnosis and up to 6 months of treatment was estimated at 97% coverage for the 110,453 patients registered in the NPCR between 1998 and 2010, all of whom were linked to the Cause of Death registry for survival outcomes [19, 20]. Follow-up data for 5 years, however, covered only roughly 69% of a 10,311 patient subgroup (by age and tumour stage) diagnosed in 2003–2005, and many specific variables (in comparison to administrative data alone) for measuring outcomes were found for this group. Similarly, PROMs to collect at baseline and after 1 year, were available for analysis for less than 5000 men (1348 collected during 2015) at publication in 2017 [18]. Noting the difficulties in collecting follow-up registry information (where many clinicians fail to send updates), the PCBaSe^Traject^ was created for more than 100,000 patients to study changes in disease and treatment patterns, though most follow-up data is still from administrative sources [20].

The list of observational studies retrieved from ClinTrials.gov examined here also revealed several examples of observational follow-up studies conducted on groups of patients identified in completed clinical trials or through prospective observational studies using established or newly formed clinical networks. Such studies, though decidedly valuable for including clinical measures, were excluded from our mapping exercise as they tend to involve smaller numbers of patients (usually less than 1000) and more limited geographical areas, concerns often cited for clinical trials in comparison to potential large-scale RWD use [3, 4].

### Limitations

This work presents several limitations. First, only the COMET database was searched, thus relying on the COMET search strategies accuracy in the identification of eligible studies. Second, the study quality assessment was performed using the COS-STAD framework, which is not strictly speaking a critical appraisal tool; as specified by the authors, researchers wishing to appraise and adopt published COS “will need to use their own judgment regarding the applicability of the COS for the purpose they require” [7]. In relation to this, one should also acknowledge the poor reporting of those studies, something that is however improving over time and through diffusion and uptake of proper guidelines. Third, several limitations affected the ‘mapping’ exercise. The assessment of COS in administrative data was limited to three types of data (hospital discharge, medication purchases, mortality registers) from five European nations, and for patient registries was based on one detailed example from Sweden that linked the registry with administrative data, with additional comparisons to other countries and data sources in more general terms. Selecting the most comprehensive national-registry in Europe with established links to relevant administrative databases in place was deemed to provide the best coverage with respect to core outcomes of interest and our estimate of overlap between COS and outcomes included in RWD sources is therefore unlikely to be representative of all RWD sources. Moreover, difficulties regarding the availability of data and reliability of coding and variables for the mapping exercise have been mentioned; additionally, full application of the methodology requires further identification of all relevant codes (beyond our one sample code) and, importantly, validation by specialized clinical personnel for code identification, which was not available for this exercise. Access to datasets also varies widely from country to country. In the EU-funded projects used as the basis for this mapping exercise (www.eurohope.info, www.bridge-health.eu), each country was required to request access to national or regional databases, a process which took considerable time and effort, and not all databases (or years) were available to each country; hence, we limited the exercise to five countries and, regarding registries, we limited the exercise to the PCBaSe, which provided considerable supporting information on websites and in published descriptions [18–20]. It is worth noting, however, that access to PCBaSe is restricted, according to the website. In this study, we encountered considerable difficulty in mapping COS for patient registries and/or registries linked with administrative data for countries other than Sweden (PCBaSe). From the registry review [17], studies in the ClinTrials.gov search, and additional literature searches, it is apparent that several other sources of registries linked to administrative data compare favourably with the PCBaSE, such as the Prostate Cancer Clinical Registry in Norway and the SEER-Medicare database in the US (which covers about 34% of the US population, https://seer.cancer.gov) [17, 42]. Various studies using the latter have analysed incidence, treatment and outcomes, including skeletal events [17, 34, 36]. SEER data is easily obtained, while SEER-Medicare linked data is restricted to investigators for specific research questions, and at least some costs are involved. SEER linked to the Medicare Health Outcomes Survey has been used for PROMs, though questions relevant to prostate cancer are limited, and the data only cover managed care plan patients [43]. The use of claims databases in the US other than the reasonably available Medicare [44], as well as the availability of EHRs in the US, were beyond the geographic scope set for this study. Finally, we considered but found it too difficult to map EMR/EHRs, as we could not find examples in use in the five European nations chosen for mapping. We assumed an EMR to be defined as “observational data from clinical practice”, including laboratory and diagnostic results and prescribed medication [45], while an EHR summarizes the ongoing health issues of a single person, linking the EMR with information from other databases, such as claims data with diagnostic and procedure codes as well as cost information. In recent years, some studies from the US have explored using EHR data to measure outcomes (most often in combination with claims data), including a Stanford University research group in California, testing the use of EHRs specifically for prostate cancer [45–47]. However, poor data quality (including accuracy of clinical coding, which is prone to subjectivity, variability and error), issues regarding privacy, ownership and access, the use of different software systems across health care settings, and the difficulty and expense of mining clinical notes may limit their application [3, 45, 46, 48].

### Future research

To our knowledge, this scoping review represents the first attempt to understand whether and how COS developed for a specific disease condition in clinical research or clinical practice can be measured through sources available and reflective of real-world practice. Overall, high-quality studies that follow the recent recommendations in terms of COS development and reporting are needed. In order to increase the uptake of COS [49, 50], their use may be mandated by research funders, trial registries, journal editors and policy-making agencies, together with better communication and collaboration among different initiatives to ensure standards align across contexts [51]. Moreover, the inclusion of PROs reflecting patient preferences, subjective symptoms, and health-related quality of life should be encouraged in COS and RWD sources developed for cancer settings (e.g., cancer registries) by taking advantage of recently booming electronic and mobile health solutions [52]. The use of PROMs in clinical practice is known to be low and fragmented, and it is documented in few places or in pockets of excellence. However, the current technological landscape would allow for a wide-scale, standardized, continuous collection of PROMs that is integrated in clinical practice and everyday care [53]. Of course issues of interoperability, data governance, security, privacy, logistics and ethics must be addressed in advance but incorporation in routinely collected data of the voice, preferences, and experience of the patient is theoretically possible locally, regionally, and even nationally.

While a promising degree of overlap between COS and RWD is present, this review and related data mapping exercise reveal that additional effort should be made to facilitate integration and cross-linkage among different databases to cover greater numbers of patients. Funding, international collaborations, and opportunities to share individual patient data from several registries should be encouraged. For example, the Cross-Border PAtient REgistries iNiTiative (PARENT), a Joint Action under the EU Health Programme 2008–2013, aims at supporting member states in developing comparable and coherent patient registries, to better enable analysis of secondary data for public health and research purposes (https://www.eunethta.eu/parent/). Many studies found in ClinTrials.gov refer to prospective patient registry creation or propose follow-up analyses of patients previously enrolled in clinical trials, allowing for measurement of important outcomes and should be further studied and developed; at least one of these (IRONMAN, https://ironmanregistry.org/) proposes to establish an international cohort registry from eight countries to study practice patterns.

## Conclusions

The growing amount of data arising from administrative systems, EHRs, registries, and other sources, represents a unique opportunity to gain insights on the comparative effectiveness and cost-effectiveness of treatments, including surgical procedures, medicines, devices, and other health technologies. Although they may have been originally developed for other purposes, the routine collection of data in real-world conditions emphasizes the need to enrich them with COS recording in order to also make them valuable for comparative effectiveness evaluation. As Sean Tunis, Senior Strategic Advisor at Center for Medical Technology Policy, recently said, “[…] *a major challenge is that the outcomes that matter most to patients are often not collected as part of RWD […] we need to work out how to get that data, rather than allowing what is most feasible to collect dominate what is most meaningful”* [54]. The combination of different data sources together with interoperability of systems is key to exploit the full potential of routinely collected data and extend the list of COS that can be captured through them, with the final aim of improving the assessment of healthcare technologies, services and outcomes for patients.

## Supplementary information

**Additional file 1 **: **Table S1.** Administrative data sets in selected European countries (i.e., Finland, Norway, Sweden, Hungary, and Italy).

## Data Availability

The research papers analysed during the current study are publically available in the COMET database [http://www.comet-initiative.org/].

## Abbreviations

ADT: Androgen Deprivation Therapy
ATC: Anatomical Therapeutic Chemical
COMET: Core Outcome Measures in Effectiveness Trials
COS: Core Outcome Set
COS-STAD: COS-STAndards for Development
COS-STAR: COS-STAndards for Reporting
EHRs: Electronic Health Records
EMRs: Electronic Medical Records
ICD10: International Classification of Diseases, 10th Version
ICD9: International Classification of Diseases, Ninth Version
NPCR: National Prostate Cancer Register
OMIs: Outcome Measurement Instruments
PCBaSe: Prostate Cancer data Base Sweden
PPC: Patient-overview Prostate Cancer
PROMs: Patient-Reported Outcome Measures
PROs: Patient-Reported Outcomes
PSA: Prostate-Specific Antigen
RWD: Real-World Data
SEER: Surveillance, Epidemiology, and End Results
US: United States
