# Supplementary material for: A scoping review of core outcome sets and their ‘mapping’ onto real-world data using prostate cancer as a case study
Source: BMC Med Res Methodol. 2020 Feb 27;20:41. doi: 10.1186/s12874-020-00928-w (PMC7045588; doi:10.1186/s12874-020-00928-w)
Supplement: Supplementary file 1 — Additional file 1 : Table S1. Administrative data sets in selected European countries (i.e., Finland, Norway, Sweden, Hungary, and Italy). [file 12874_2020_928_MOESM1_ESM.docx]

**Table S1. Administrative data sets in selected European countries (i.e., Finland, Norway, Sweden, Hungary, and Italy).**

|  | **Data source** | **Geographical coverage** | **Demographics (individual level)** | **Clinical/Coding** | **Resource use** | **Outcomes** |
| --- | --- | --- | --- | --- | --- | --- |
| **HOSPITALIZATION** | | | | | | |
| **Finland** | Care Register for Health Care  (inpatient and day hospital) | National | Age  Gender  Residency | Diagnoses (ICD10-CM codes since 1996, ICD9-CM from 1986-1996)  Procedures (Finnish version of NOMESCO procedure classification system)  Discharge status | Length of stay (day)  Hospitalization type (inpatient admission vs day surgery, surgical vs medical, etc.)  Costs (DRG) | Hospitalizations  Intra-hospital mortality  Length of stay  Hospitalization costs  Symptoms, comorbidities (diagnosis codes)  Procedures (procedure codes) |
| **Norway** | Norwegian register of patient records | National | Age  Gender  Residency | Diagnoses (ICD10-CM codes)  Procedures (bridge coding between the Nordic Classification of Surgical Procedures (NCSP) and ICD9  Discharge status | Length of stay (day)  Hospitalization type (inpatient admission vs day surgery, surgical vs medical, etc.)  Costs (DRG) | Hospitalizations  Intra-hospital mortality  Length of stay  Hospitalization costs  Symptoms, comorbidities (diagnosis codes)  Procedures (procedure codes) |
| **Sweden** | Swedish National Inpatient Registry (IPR) | National | Age  Gender  Residency | Diagnoses (ICD-10)  Procedures (national classification of health interventions or KVÅ, a Swedish version of the NOMESCO system)  Discharge status | Length of stay (day)  Hospitalization type (inpatient admission vs day surgery)  Costs (DRG) | Hospitalizations  Intra-hospital mortality  Length of stay  Hospitalization costs  Symptoms, comorbidities (diagnosis codes)  Procedures (procedure codes) |
| **Hungary** | Hospital Discharge Register (HDR), National Health Insurance Fund Administration of Hungary | National | Age  Gender  Residency | Diagnoses (ICD10-CM codes)  Procedures (country specific coding system)  Discharge status | Length of stay (day)  Hospitalization type (inpatient admission vs day surgery, surgical vs medical, etc.)  Costs (DRG) | Hospitalizations  Intra-hospital mortality  Length of stay  Hospitalization costs  Symptoms, comorbidities (diagnosis codes)  Procedures (procedure codes) |
| **Italy** | Hospital Discharge Database (SDO) | Regional (national SDO database is not linkable to other databases) | Age  Gender  Residence (Region/District/Municipality) | Diagnoses (ICD9-CM codes)  Procedures (ICD9-CM procedure codes)  Discharge status | Length of stay (day)  Hospitalization type (inpatient admission vs day surgery, surgical vs medical, etc.)  Costs (DRG)  Payer (e.g., national health system, patient, other) | Hospitalizations  Intra-hospital mortality  Length of stay  Hospitalization costs  Symptoms, comorbidities (diagnosis codes)  Procedures (procedure codes) |

**Table S1. (**cont.)

|  | **Data source** | **Geographical coverage** | **Demographics (individual level)** | Clinical/Coding | **Resource use** | **Outcomes** |
| --- | --- | --- | --- | --- | --- | --- |
| **PRESCRIBED MEDICATION** | | | | | | |
| **Finland** | Prescribed medication | National | Patient ID | ATC code | Number of units  Date purchased  Cost | Medication use  Costs  Indications of comorbidities |
| **Norway** | National Database for Electronic Prescriptions | National | Patient ID | ATC code | Number of units  Date purchased  Cost | Medication use  Costs  Indications of comorbidities |
| **Sweden** | Prescription registry | National | Patient ID | ATC code | Prescriptions administered at outpatient pharmacies | Medication use  Costs  Indications of comorbidities |
| **Hungary** | Prescribed medicine, National Health Insurance Fund Administration of Hungary | National | Patient ID | ATC code | Number of units  Date purchased  Cost | Medication use  Costs  Indications of comorbidities |
| **Italy** | Publicly funded medication purchases | Regional | Patient ID | ATC code | Number of prescriptions  Date purchased  Cost | Medication use  Costs  Indications of comorbidities |
|  | Publicly funded medications administered during hospital stays | Regional | Patient ID | ATC code | Medications administered in the hospital, units, dates | Medication use  Indications of comorbidities |

**Table S1. (**cont.)

|  | **Data source** | **Geographical coverage** | **Demographics (individual level)** | **Clinical/Coding** | **Resource use** | **Outcomes** |
| --- | --- | --- | --- | --- | --- | --- |
| **MORTALITY REGISTRIES** | | | | | | |
| **Finland** | Mortality registry | National | Age  Gender | Cause of death  Date of death | NA | Cause of death  Deaths/mortality rates |
| **Norway** | Causes of Death Registry | National | Age  Gender | Cause of death  Date of death | NA | Cause of death  Deaths/mortality rates |
| **Sweden** | Causes of death registry | National | Age  Gender | Cause of death  Date of death | NA | Cause of death  Deaths/mortality rates |
| **Hungary** | Date of death statistics | National | Age  Gender | Date of death, cause of death is not available, though it is collected | NA | Deaths/mortality rates |
| **Italy** | Mortality registries (regional) | All regions | Age class  Gender | Cause of death (ICD9 codes until 2006; ICD10 thereafter)  Date of death | NA | Cause of death  Deaths/mortality rates |

ATC: Anatomical Therapeutic Chemical; CM: Clinical Modification; DRG: Diagnosis-Related Group; ICD: International Classification of Diseases; NA: not availbale.
